# Image quality and metal artifact reduction in total hip arthroplasty CT: deep learning-based algorithm *versus* virtual monoenergetic imaging and orthopedic metal artifact reduction

**DOI:** 10.1186/s41747-024-00427-3

**Published:** 2024-03-14

**Authors:** Mark Selles, Ruud H. H. Wellenberg, Derk J. Slotman, Ingrid M. Nijholt, Jochen A. C. van Osch, Kees F. van Dijke, Mario Maas, Martijn F. Boomsma

**Affiliations:** 1 Department of Radiology, Isala, 8025 AB Zwolle, the Netherlands; 2 Department of Radiology & Nuclear Medicine, Amsterdam University Medical Centre, 1105 AZ Amsterdam, the Netherlands; 3 Amsterdam Movement Sciences, 1081 BT Amsterdam, the Netherlands; 4 Department of Medical Physics, Isala, 8025 AB Zwolle, the Netherlands; 5 Department of Radiology & Nuclear Medicine, Noordwest Ziekenhuisgroep, 1815 JD Alkmaar, the Netherlands

**Keywords:** Arthroplasty (replacement, hip), Artificial intelligence, Artifacts, Deep learning, Tomography (x-ray computed)

## Abstract

**Background:**

To compare image quality, metal artifacts, and diagnostic confidence of conventional computed tomography (CT) images of unilateral total hip arthroplasty patients (THA) with deep learning-based metal artifact reduction (DL-MAR) to conventional CT and 130-keV monoenergetic images with and without orthopedic metal artifact reduction (O-MAR).

**Methods:**

Conventional CT and 130-keV monoenergetic images with and without O-MAR and DL-MAR images of 28 unilateral THA patients were reconstructed. Image quality, metal artifacts, and diagnostic confidence in bone, pelvic organs, and soft tissue adjacent to the prosthesis were jointly scored by two experienced musculoskeletal radiologists. Contrast-to-noise ratios (CNR) between bladder and fat and muscle and fat were measured. Wilcoxon signed-rank tests with Holm-Bonferroni correction were used.

**Results:**

Significantly higher image quality, higher diagnostic confidence, and less severe metal artifacts were observed on DL-MAR and images with O-MAR compared to images without O-MAR (*p* < 0.001 for all comparisons). Higher image quality, higher diagnostic confidence for bone and soft tissue adjacent to the prosthesis, and less severe metal artifacts were observed on DL-MAR when compared to conventional images and 130-keV monoenergetic images with O-MAR (*p* ≤ 0.014). CNRs were higher for DL-MAR and images with O-MAR compared to images without O-MAR (*p* < 0.001). Higher CNRs were observed on DL-MAR images compared to conventional images and 130-keV monoenergetic images with O-MAR (*p* ≤ 0.010).

**Conclusions:**

DL-MAR showed higher image quality, diagnostic confidence, and superior metal artifact reduction compared to conventional CT images and 130-keV monoenergetic images with and without O-MAR in unilateral THA patients.

**Relevance statement:**

DL-MAR resulted into improved image quality, stronger reduction of metal artifacts, and improved diagnostic confidence compared to conventional and virtual monoenergetic images with and without metal artifact reduction, bringing DL-based metal artifact reduction closer to clinical application.

**Key points:**

• Metal artifacts introduced by total hip arthroplasty hamper radiologic assessment on CT.

• A deep-learning algorithm (DL-MAR) was compared to dual-layer CT images with O-MAR.

• DL-MAR showed best image quality and diagnostic confidence.

• Highest contrast-to-noise ratios were observed on the DL-MAR images.

**Graphical Abstract:**

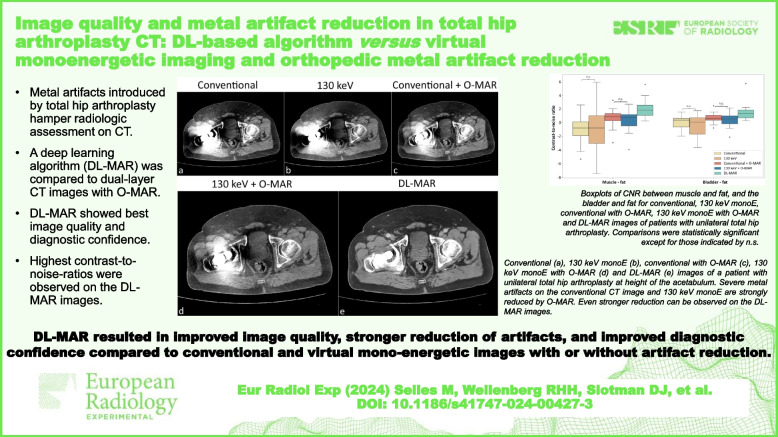

**Supplementary Information:**

The online version contains supplementary material available at 10.1186/s41747-024-00427-3.

## Background

Total hip arthroplasty (THA) is widely used as an effective treatment for pain in patients with osteoarthritis or inflammatory arthritis [1]. More than 1.8 million THA procedures are performed annually worldwide, and this number is expected to increase in the coming years [2]. Computed tomography (CT) is often the imaging modality of choice for follow-up of these patients because CT is fast, relatively inexpensive, and well suited for visualizing bone effects [3]. In addition, there is a large group of patients with THA who undergo CT scans of the pelvis or abdomen for clinical indications other than THA follow-up. However, metal hip implants introduce metal artifacts in these CT images due to scattering, beam hardening, and photon starvation effects which are typically observed as dark and bright streaking artifacts throughout the image, hampering the assessment of bone and soft tissue [4, 5].

Metal artifacts can be reduced by increasing tube voltage or by repositioning of the patient but also by more advanced reconstruction-based metal artifact reduction techniques such as orthopedic metal artifact reduction (O-MAR), which rely on the detection and replacement of projection data that is affected by metal artifacts. Another method for metal artifact reduction is the use of high-energy virtual monoenergetic images (monoE) that are useful for reduction of beam hardening artifacts and can be generated by dual energy CT or photon counting CT [5–7]. High-energy monoE are useful for the reduction of relatively mild artifacts but come at the cost of reduced overall image contrast. Although the optimal energy is dependent on the implant material, size, and shape, monoE of 130 keV are generally effective in reducing these mild metal artifacts in CT images with hip prostheses [8]. High-energy monoE are of limited use when dealing with more severe metal artifacts. In these cases, reconstruction-based metal artifact reduction techniques should be used [4, 5]. However, these reconstruction-based techniques may introduce secondary artifacts [6, 9–11].

With the emergence of deep learning (DL), numerous DL-based techniques have been developed for detection, segmentation, classification, and prediction purposes within radiology [12]. Deep learning-based metal artifact reduction techniques (DL-MAR) for CT have also shown promising results [13–17]. These models are typically trained using simulated CT data which allows for the synthesis of CT images with and without metal artifacts [13–17]. However, studies reporting on external evaluation of DL-MAR using clinical CT data are limited. Recently, a first quantitative evaluation in patients after sacroiliac joint fusion showed strong reduction of metal artifacts by a generic DL-MAR, outperforming O-MAR [18]. To date, no qualitative or quantitative evaluation of DL-MAR has been performed in THA patients and the ability of DL-MAR to reduce metal artifacts in CT images of THA patients compared to O-MAR, and high-energy monoE is unknown. Therefore, the aim of this study was to compare conventional CT with and without O-MAR, 130-keV monoE with and without O-MAR, and DL-MAR of patients with unilateral THA in terms of image quality and metal artifacts with regard to diagnostic confidence. We hypothesize that CT images corrected with DL-MAR will result in less metal artifacts, higher image quality and higher diagnostic confidence than conventional CT with and without O-MAR, and 130-keV monoE with and without O-MAR.

## Methods

### Patient population

Thirty-one consecutive patients with a unilateral hip prosthesis, scheduled for a CT scan in Isala Hospital Meppel, were prospectively included between August 2022 and April 2023. One patient had an additional metal implant in the arm and was therefore excluded for analysis. The study was approved by our institutional review board (number 20220412). The need for informed consent was waived.

### Image acquisition and reconstruction

Patients were scanned on a Philips Spectral CT 7500 system (Philips Healthcare, Best, the Netherlands) (Table 1). Since patients were consecutively included for varying indications, slightly different scan protocols were used. Conventional polychromatic images and 130-keV monoE of the 30 patients were reconstructed with and without O-MAR. In addition, a generic DL-MAR was applied to the conventional image without O-MAR. Details about the development of this DL-MAR algorithm such as data, ground truth, data partitioning, model, training, and evaluations are described in a previous study [18]. In summary, the DL-MAR algorithm was developed using simulated data, which allowed for the synthesis of paired CT images containing metal implants with and without metal artifacts. A total of 105,163 paired images were used to train the DL-MAR algorithm using a deep residual U-NET architecture. The network was trained using a combined loss function existing in a SSIM loss, L1 loss, and L2 loss.

**Table 1:** Acquisition and reconstruction parameters

Parameter	Value
Tube voltage	
120 kVp (number)	26
140 kVp (number)	4
Exposure (mAs)	10.2 ± 3.9
CTDI_vol_ (mGy)	128.2 ± 54.5
Slice thickness (mm)	1
Increment (mm)	0.5
Rotation time (s)	0.4
Image matrix (pixels)	512 × 512
Collimation (mm)	128 × 0.625
Filter	Sharp

Data of exposure and CTDI_vol_ are given as mean ± standard deviation. *CTDI* Volumetric computed tomography dose index

### Subjective evaluation

All conventional images with and without O-MAR, 130-keV monoE with and without O-MAR, and the DL-MAR images were jointly assessed by two radiologists from two different hospitals to the one where DL-MAR was developed (C.F.v.D., 25 years of experience; M.M., 28 years of experience) to reach consensus per reconstruction per patient on the following questions:

1. How would you rate image quality in general?
2. How would you rate the diagnostic confidence for bone structures?
3. How would you rate the diagnostic confidence for pelvic organs?
4. How would you rate the diagnostic confidence for soft tissue adjacent to the prosthesis?
5. How would you rate the metal artifacts in the image?

For questions 1–4, a 5-point scale with a score from 1 to 5 was used, including very poor (1), poor (2), fair (3), good (4), and excellent (5). For question 5, a 5-point scale including severe (1), pronounced (2), moderate (3), mild (4), and none (5) was used. The CT images of two patients were used for a training session of the radiologists, and the CT images of the other 28 patients were used for the actual qualitative scoring.

MeVisLab (MeVis Medical Solutions AG, Fraunhofer MEVIS, Bremen, Germany) was used to develop an interface to present the anonymized CT images in random order alongside the scoring form. The radiologists assessed CT images in axial view in bone window (width 1,600, level 400 HU) and soft tissue window (400 and 40 HU, respectively).

### Objective evaluation

**Fig. 1:**
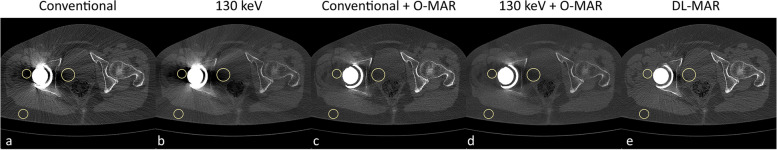
Conventional (**a**), 130-keV monoE (**b**), conventional with O-MAR (**c**), 130-keV monoE with O-MAR (**d**), and DL-MAR (**e**) images of a patient with unilateral total hip arthroplasty at the middle of the prosthesis’ head. Three regions of interest were drawn on the conventional CT image and subsequently copied to the other reconstructed images to measure CT values and noise as the standard deviation in HU. Window: width 1,600, level 400 HU. *CT* Computed tomography, *O-MAR* Orthopedic metal artifact reduction, *DL-MAR* Deep learning-based metal artifact reduction

Three regions of interest (ROIs) were drawn by an experienced researcher (MS) on the conventional images without O-MAR on the axial slice at the middle of the prosthesis’ head using the “Fiji” open-source platform [19]. One circular ROI with a diameter of 30 mm was placed in the bladder at the medial side of the hip prosthesis. Another circular ROI with a diameter of 19 mm was placed at the lateral side of the hip prosthesis in muscle in the area with most pronounced artifacts. A third circular ROI with a diameter of 19 mm was placed in gluteal subcutaneous fat at the side of the hip prosthesis. Only in patients with a relatively thin gluteal subcutaneous fat the diameter of this ROI was carefully resized to assure that only fat was measured. Figure 1 shows the locations where the ROIs were placed (see supplemental materials 1 for specific examples). The ROIs were copied to the exact same locations on the other reconstructed images for each patient. Mean CT values in Hounsfield units (HU) and noise as the standard deviation in HU were measured in all ROIs. Contrast-to-noise ratios (CNRs) between the bladder and fat and between muscle and fat were calculated by dividing the difference in CT values between the tissues by the mean noise values of both tissues. To assess interobserver reliability of ROI placement, ROIs were also placed in the bladder, muscle, and fat on the conventional images without O-MAR of 15 randomly selected patients by an experienced radiologist (M.F.B., 14 years of experience).

### Statistical methods

The scores on the questionnaire were considered as continuous variables and analyzed for each question separately. The eyeball test in combination with the Shapiro-Wilk test showed that the data was not normally distributed. Wilcoxon signed-rank tests were used to test for differences between conventional images, conventional images with O-MAR, 130-keV monoE, 130-keV monoE with O-MAR, and DL-MAR. Adjusted *p*-values were calculated using the Holm-Bonferroni method to adjust for multiple comparisons. A significance level of 5% was used. In addition, the interobserver reliability between the two observers was calculated using the intraclass correlation coefficient for absolute agreement using a two-way mixed model. IBM SPSS software Version 27.0 (IBM Corporation, Armonk, NY, USA) was used for all statistical analyses.

## Results

### Subjective evaluation

Lowest scores were observed for the conventional images without O-MAR and 130-keV monoE images without O-MAR on the questions concerning image quality, diagnostic confidence for bone, diagnostic confidence for pelvic organs, diagnostic confidence for soft tissue adjacent to the prosthesis, and metal artifacts (Table 2).

**Table 2:** Median and interquartile range (IQR) for conventional images, 130-keV monoE, conventional images with O-MAR, 130-keV monoE with O-MAR, and DL-MAR images

	Conventional (median, IQR)	130 keV	Conventional + O-MAR	130 keV + O-MAR	DL-MAR
Image quality	1 (1–2)	1 (1–2)	2 (2–3)^a^	2 (2–3)^a^	3 (3–4)^b^
Diagnostic confidence					
Bone	1 (1–1)	1 (1–2)	2 (2–3)^a^	2 (2–3)^a^	4 (3–4)^b^
Pelvic organs	1 (1–2)	1 (1–1)	2 (2–3)^a^	2 (1–3)^a^	4 (2–4)^a^
Soft tissue adjacent to the prosthesis	1 (1–2)	1 (1–2)	2 (2–3)^a^	2 (2–3)^a^	3 (3–4)^b^
Metal artifacts	1 (1–1)	1 (1–1)	2 (2–3)^a^	2 (2–3)^a^	3 (3–4)^b^

*IQR* Interquartile range, *O-MAR* Orthopedic metal artifact reduction, *DL-MAR* Deep learning-based metal artifact reduction

^a^Significant reduction in comparison to conventional images and 130-keV monoE

^b^Significant reduction in comparison to conventional images, 130-keV monoE, conventional images with O-MAR, and 130-keV monoE with O-MAR

The two radiologists scored image quality; diagnostic confidence for bone, pelvic organs, and soft tissue adjacent to the prosthesis; and metal artifacts significantly higher on conventional images with O-MAR and 130-keV monoE with O-MAR than on conventional images and 130-keV monoE without O-MAR (*p* ≤ 0.001 for all comparisons; see supplemental materials 2 for *p*-values of all pairwise comparisons). DL-MAR was also rated significantly higher on image quality, diagnostic confidence in all studied areas, and metal artifacts in comparison to conventional images without O-MAR and 130-keV monoE without O-MAR (*p* < 0.001).

**Fig. 2:**
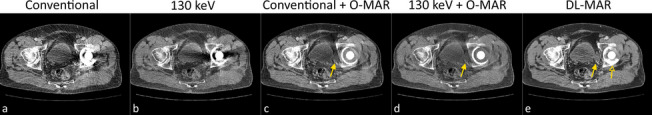
Conventional (**a**), 130-keV monoE (**b**), conventional with O-MAR (**c**), 130-keV monoE with O-MAR (**d**), and DL-MAR (**e**) images of a patient with unilateral total hip arthroplasty at level of the acetabulum. Artifacts on the conventional CT image and 130-keV monoE conceal a bladder cyst to the right of the prosthesis cup that is visible on the conventional image O-MAR, 130-keV monoE with O-MAR, and DL-MAR (solid yellow arrow). Although not observed in all patients, some artifacts remain between the prosthesis cup and prosthesis head on DL-MAR (open yellow arrow). Window: width 400, level 40 HU. *CT* Computed tomography, *O-MAR* Orthopedic metal artifact reduction, *DL-MAR* Deep learning-based metal artifact reduction

**Fig. 3:**
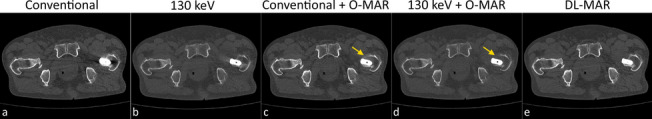
Conventional (**a**), 130-keV monoE (**b**), conventional with O-MAR (**c**), 130-keV monoE with O-MAR (**d**), and DL-MAR (**e**) images of a patient with unilateral total hip arthroplasty at height of the prosthesis’ femoral stem. In this patient, metal artifacts are reduced on the images with O-MAR, but secondary artifacts are introduced resulting in degradation of cortical bone anterior to the femoral stem (yellow arrow). DL-MAR reduces artifacts without introducing these secondary artifacts. Window: width 1,600, level 400 HU. *CT* Computed tomography, *O-MAR* Orthopedic metal artifact reduction, *DL-MAR* Deep learning-based metal artifact reduction

**Fig. 4:**
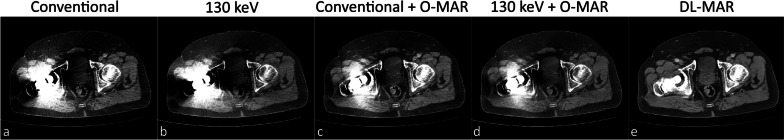
Conventional (**a**), 130-keV monoE (**b**), conventional with O-MAR (**c**), 130-keV monoE with O-MAR (**d**), and DL-MAR (**e**) images of a patient with unilateral total hip arthroplasty at height of the acetabulum. Severe metal artifacts on the conventional CT image and 130-keV monoE that are strongly reduced by O-MAR. Even stronger reduction can be observed on the DL-MAR images. Window: width 400, level 40 HU. *CT* Computed tomography, *O-MAR* Orthopedic metal artifact reduction, *DL-MAR* Deep learning-based metal artifact reduction

**Fig. 5:**
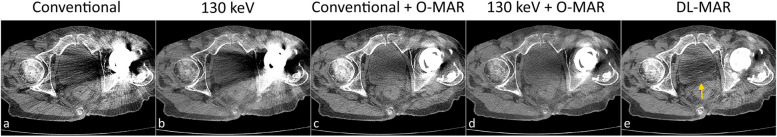
Conventional (**a**), 130-keV monoE (**b**), conventional with O-MAR (**c**), 130-keV monoE with O-MAR (**d**), and DL-MAR (**e**) images of a patient with unilateral total hip arthroplasty at height of the acetabulum. Although not observed in all patients, some artifacts are remaining in the lesser pelvis on the DL-MAR image (yellow arrow). *O-MAR* Orthopedic metal artifact reduction, *DL-MAR* Deep learning-based metal artifact reduction

Responses to questions on image quality, diagnostic confidence for bone, diagnostic confidence for soft tissue adjacent to the prosthesis, and metal artifacts on DL-MAR were significantly higher than on conventional images with O-MAR and 130-keV monoE with O-MAR (*p* ≤ 0.014 for all comparisons; Figs. 2, 3 and 4). No significant difference was observed when comparing scores for diagnostic confidence for pelvic organs on DL-MAR to conventional images with O-MAR (*p* = 0.063; Fig. 5) and 130-keV monoE with O-MAR (*p* = 0.013, but not significant after Holm-Bonferroni correction).

No statistical differences between were observed when comparing the radiologists’ scores on conventional images without O-MAR to 130-keV monoE without O-MAR for image quality, diagnostic confidence for bone, pelvic organs and soft tissue adjacent to the prosthesis, and metal artifacts. Also, no significant differences were observed when comparing conventional images with O-MAR to 130-keV monoE with O-MAR for image quality, diagnostic confidence in all studied areas, and metal artifacts.

### Objective evaluation

An intraclass correlation coefficient of 0.841 was calculated for CT values measured in ROIs placed in the bladder, indicating good interobserver reliability [20]. For ROIs placed in muscle and fat, intraclass correlation coefficients of 0.960 and 0.944, respectively, were observed indicating excellent interobserver agreement [20].

**Fig. 6:**
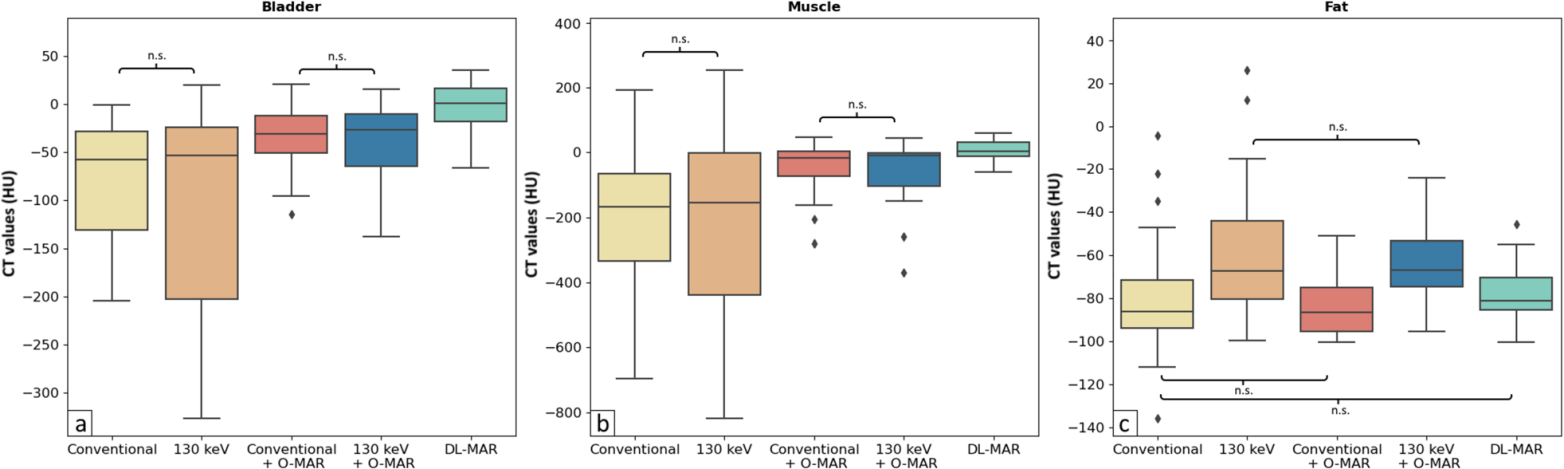
Boxplots of HU in the bladder (**a**), muscle (**b**), and fat (**c**) for conventional, 130-keV monoE, conventional with O-MAR, 130-keV monoE with O-MAR, and DL-MAR images of patients with unilateral total hip arthroplasty. All comparisons between reconstructions were statistically significant (*p* ≤ 0.05) except for those indicated by n.s. *O-MAR* Orthopedic metal artifact reduction, *DL-MAR* Deep learning-based metal artifact reduction

HU values of the bladder and muscle were significantly higher on conventional images with O-MAR and 130-keV images with O-MAR compared to conventional images and 130-keV images without O-MAR (*p* < 0.002 for all comparisons; Fig. 6; see supplemental materials 3 for *p*-values of all pairwise comparisons). DL-MAR images showed significantly higher HU values of the bladder and muscle compared to all other reconstructions (*p* < 0.001 for all comparisons). HU values of fat were highest on 130-keV monoE with and without O-MAR compared to the other reconstructions (*p* < 0.002 for all comparisons). HU values of fat on DL-MAR images were significantly higher compared to conventional images with O-MAR (*p* = 0.004).

**Fig. 7:**
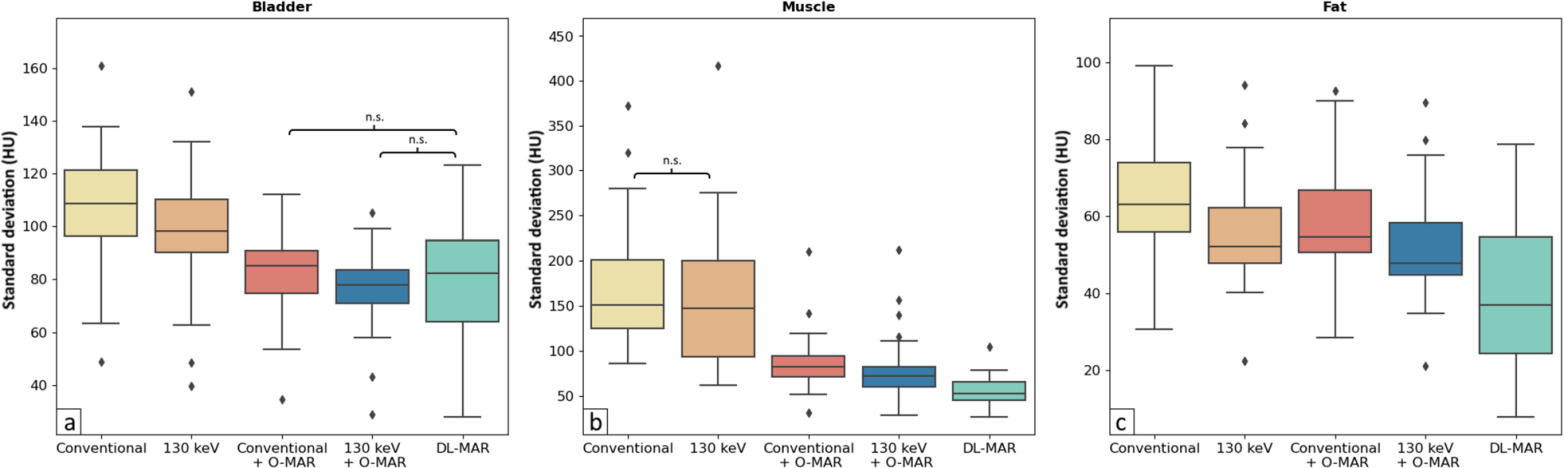
Boxplots of noise as the standard deviation in HU in the bladder (**a**), muscle (**b**), and fat (**c**) for conventional, 130-keV monoE, conventional with O-MAR, 130-keV monoE with O-MAR, and DL-MAR images of patients with unilateral total hip arthroplasty. All comparisons between reconstructions were statistically significant (*p* ≤ 0.05) except for those indicated by n.s. *O-MAR* Orthopedic metal artifact reduction, *DL-MAR* Deep learning-based metal artifact reduction

Noise in the bladder and fat was lower on 130-keV monoE without O-MAR compared to conventional images without O-MAR (*p* < 0.001 for all comparisons; Fig. 7). Conventional images with O-MAR showed lower noise in the bladder and muscle compared to conventional images without O-MAR and 130-keV images without O-MAR (*p* < 0.002 for all comparisons). Noise in fat was lower on conventional images with O-MAR compared to conventional images without O-MAR (*p* < 0.001), while noise in fat was higher on conventional images with O-MAR compared to 130 keV without O-MAR (*p* < 0.001). Noise values in the bladder, muscle, and fat were lower on 130-keV monoE with O-MAR compared to conventional images with and without O-MAR and 130-keV monoE without O-MAR (*p* < 0.002 for all comparisons). DL-MAR showed lower noise in the bladder compared to conventional images without O-MAR and 130-keV monoE without O-MAR (*p* < 0.001 for all comparisons) and lower noise in muscle and fat than all other reconstructions (*p* < 0.002 for all comparisons).

**Fig. 8:**
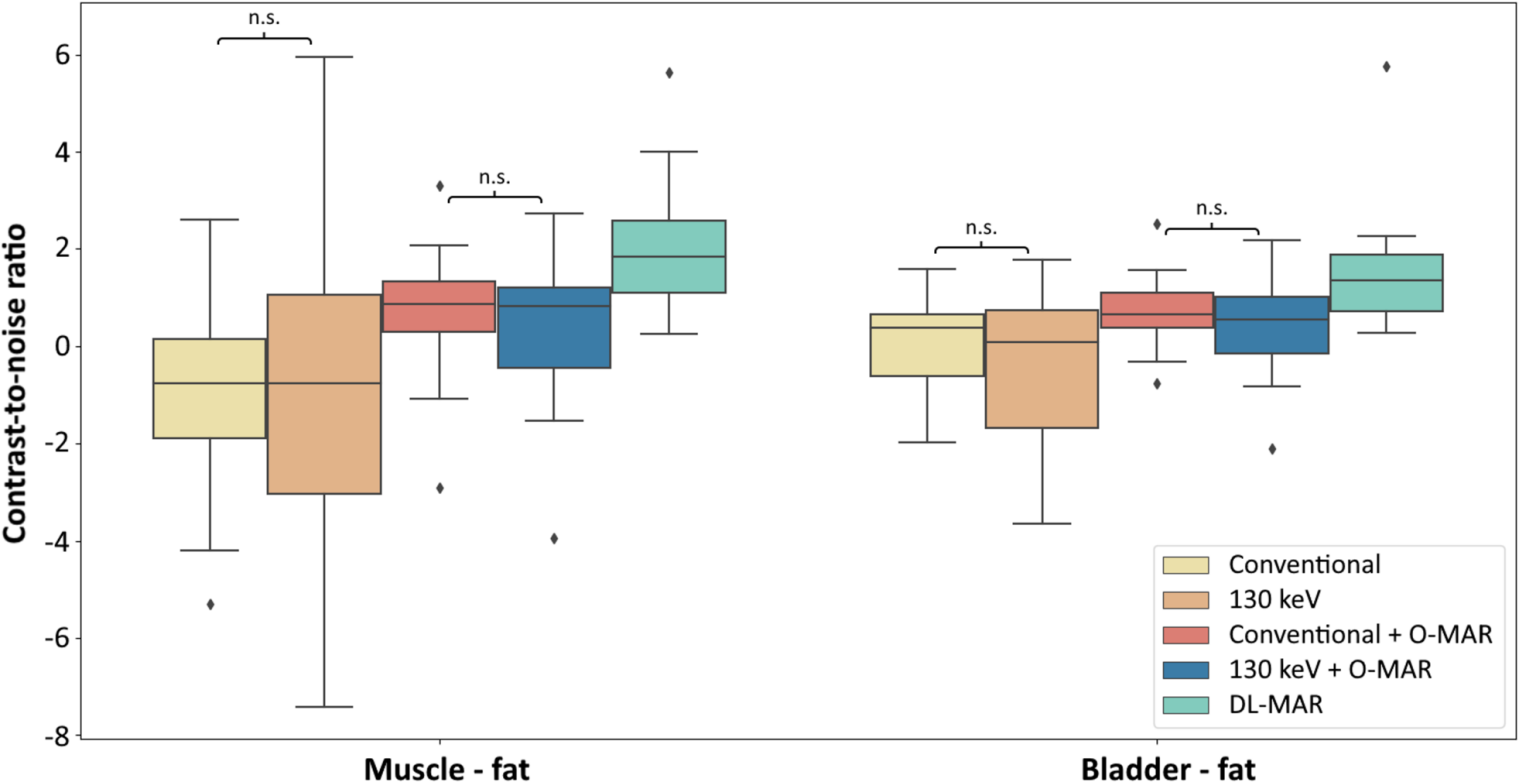
Boxplots of contrast-to-noise ratios (CNR) between muscle and fat and the bladder and fat for conventional, 130-keV monoE, conventional with O-MAR, 130-keV monoE with O-MAR, and DL-MAR images of patients with unilateral total hip arthroplasty. All comparisons between reconstructions were statistically significant (*p* ≤ 0.05) except for those indicated by n.s. *O-MAR* Orthopedic metal artifact reduction, *DL-MAR* Deep learning-based metal artifact reduction

CNR between muscle and fat and CNR between the bladder and fat were significantly higher on conventional images with O-MAR than on conventional images without O-MAR and 130-keV monoE without O-MAR (*p* < 0.001; Fig. 8). Monochromatic 130-keV images with O-MAR showed a significantly higher CNR between muscle and fat compared to conventional images without O-MAR (*p* = 0.003) and 130-keV monoE without O-MAR (*p* = 0.005). Monochromatic 130-keV images with O-MAR also showed a significantly higher CNR between the bladder and fat compared to conventional images without O-MAR (*p* = 0.016) and 130-keV monoE without O-MAR (*p* = 0.002).

CNR between muscle and fat was significantly higher on DL-MAR images compared to all other reconstructed images (*p* < 0.001 for all comparisons). CNR between the bladder and fat was also significantly higher on DL-MAR compared to conventional images without O-MAR (*p* < 0.001), 130-keV monoE without O-MAR (*p* < 0.001), conventional images with O-MAR (*p* = 0.010), and 130-keV monoE with O-MAR (*p* < 0.001). No significant difference in CNR was observed when comparing conventional images without O-MAR to 130-keV monoE without O-MAR and when comparing conventional images with O-MAR to 130-keV monoE with O-MAR.

## Discussion

In this study, we compared a novel generic DL-MAR algorithm for the reduction of metal artifacts to an established metal artifact reduction technique, O-MAR, in CT scans with unilateral THA. Strongest reduction of metal artifacts, highest image quality, and highest diagnostic confidence in bone and soft tissue adjacent to the prosthesis were observed on the DL-MAR images compared to conventional images and 130-keV monoE with or without O-MAR. This study is the first to perform a prospective evaluation of DL-MAR in patients with unilateral THA, thereby moving the field of DL-based MAR closer to the clinical application of deep learning-based methods for metal artifact reduction.

Although DL-MAR can greatly reduce metal artifacts resulting in improved diagnostic confidence and image quality, some metal artifacts were still present. In particular, metal artifact reduction was not shown consistently in the lesser pelvis on DL-MAR images. This may explain why there was no significant difference in diagnostic confidence for pelvic organs between DL-MAR images and conventional images with O-MAR and 130-keV monoE with O-MAR. However, the lack of a significant difference may also be due to the relatively small study population. In particular, severe metal artifacts sometimes remain present on DL-MAR images, for example, between the cup and head of the prosthesis. This may be explained by the fact that the conventional image is used as the input for DL-MAR. When artifacts on the conventional image are so severe that they completely obscure anatomical structures, then there is no information in the conventional image available that DL-MAR can use to reduce metal artifacts. In contrast to DL-MAR, O-MAR is able to depict the prosthesis cup accurately, probably because O-MAR has access to the projection data. This indicates what has already been suggested in simulation studies, i.e., that the use of projection data may further improve the ability of DL-MAR to reduce metal artifacts [13, 14, 21–23]. However, the development and clinical application of such an algorithm are challenging as this requires the cooperation of CT vendors to provide access to the projection data.

Conventional images with O-MAR were rated significantly higher by two experienced radiologists in terms of image quality, diagnostic confidence, and reduction of metal artifacts compared to conventional images without O-MAR, which is consistent with results of previous studies [24–29]. Our finding that 130-keV images with O-MAR were of higher image quality, diagnostic confidence showed stronger reduction of metal artifacts in comparison to 130-keV image without O-MAR is also in line with previous studies [6, 29, 30]. We did not find statistical differences between 130-keV monoE with O-MAR and conventional images with O-MAR or 130-keV monoE without O-MAR and conventional images without O-MAR, while several previous studies did report a significant difference between these images [27, 29, 30]. Laukamp et al. found stronger reduction of metal artifacts and improved visibility of bone, muscle, and pelvic organs when monoE of 110, 150, and 200 keV were compared to conventional images [27]. Yoo et al. found improved visibility of periprosthetic bone when comparing a set of multiple high-energy monoE ranging from 50 to 200 keV to conventional images [29]. Furthermore, Neuhaus et al. observed stronger reduction of metal artifacts and improved visibility of pelvic organs and bone in 180- and 200-keV monoE images with O-MAR compared to conventional images with O-MAR [30]. The discrepancy between the results of our study and the studies of Laukamp et al. [27], Yoo et al. [29], and Neuhaus et al. [30] may be explained by the fact that they included patients with bilateral prostheses that introduce more severe metal artifacts than the unilateral prostheses included in our study.

Studies comparing DL-based metal artifact reduction with widely used metal artifact reduction techniques are scarce. One quantitative study reported stronger reduction of metal artifacts by DL-MAR compared to O-MAR in patients scanned before and after sacroiliac joint fusion [18]. Other studies investigating DL-based metal artifact reduction focused on development of the algorithm rather than clinical evaluation. They typically evaluated one or two cases without comparison to commercial metal artifact reduction techniques or high-energy monoE [13, 16, 17, 22, 31–33]. Further studies are needed to evaluate the potential of DL-MAR in clinical practice, preferably using paired pre-surgery and post-surgery CT-images with and without metal because CT images without metal provide the most optimal reference within patients. These studies should focus not only on THA or sacroiliac joint fusion but also on other implants because DL-MAR was trained on a wide variety of metal implants. Furthermore, future research focuses on individual pathologies to investigate the clinical utility of DL-MAR.

This study has several limitations. First, the shape, size, and material of hip prosthesis affects the severity of metal artifacts [5, 8]. However, to investigate this further would require a much larger cohort of patients and, given our prospective design, a much longer inclusion period. We chose to analyze consecutive patients which reflect the presentation of hip prosthesis in CT images in clinical practice. Second, the images were scored by two radiologists, while scoring by more radiologists could be a more comprehensive approach. However, the differences in image quality and metal artifacts between the reconstructions studied are large, and both radiologists were experienced MSK radiologists in the assessment of CT images with metal artifacts. Therefore, we expect similar results if more radiologists were asked to evaluate the images. Finally, the radiologists assessed the images in axial view, whereas the coronal and sagittal views are also provided in clinical practice. However, we expect similar results if the other views had been provided because the image quality, metal artifacts, and diagnostic confidence for bone, pelvic organs, and soft tissue adjacent to the prosthesis could be assessed very well on the axial images.

Based on our results, we conclude that DL-MAR yields superior image quality and diagnostic confidence, and strongest reduction of metal artifacts compared to conventional images and 130-keV monoE with or without O-MAR in patients with unilateral THA.

### Supplementary Information

**Additional file 1: Supplemental materials 1.** Three examples (a-c) of placement of regions of interest (ROI) in the bladder (ROI 1), muscle (ROI 2) and fat (ROI 3). **Supplemental materials 2A.** *P*-values for pairwise comparisons of the reconstructed images for image quality. *statistically significant after Holm-Bonferroni correction. Abbreviations: keV: kiloelectron volt, O-MAR: orthopedic metal artifact reduction, DL-MAR: deep learning based metal artifact reduction. **Supplemental materials 2B**. *P*-values for pairwise comparisons of the reconstructed images for diagnostic confidence for bone structures.*statistically significant after Holm-Bonferroni correction. Abbreviations: keV: kiloelectron volt, O-MAR: orthopedic metal artifact reduction, DL-MAR: deep learning based metal artifact reduction. **Supplemental materials 2C.** *P*-values for pairwise comparisons of the reconstructed images for diagnostic confidence for pelvic organs.*statistically significant after Holm-Bonferroni correction. Abbreviations: keV: kiloelectron volt, O-MAR: orthopedic metal artifact reduction, DL-MAR: deep learning based metal artifact reduction. **Supplemental materials 2D.** *P*-values for pairwise comparisons of the reconstructed images for diagnostic confidence for soft tissue adjacent to the prosthesis. *statistically significant after Holm-Bonferroni correction. Abbreviations: keV: kiloelectron volt, O-MAR: orthopedic metal artifact reduction, DL-MAR: deep learning based metal artifact reduction. **Supplemental materials 2E.** *P*-values for pairwise comparisons of the reconstructed images for metal artifacts. *statistically significant after Holm-Bonferroni correction. Abbreviations: keV: kiloelectron volt, O-MAR: orthopedic metal artifact reduction, DL-MAR: deep learning based metal artifact reduction. **Supplemental materials 3A.** *P*-values for pairwise comparisons of Hounsfield units in bladder. *statistically significant after Holm-Bonferroni correction. Abbreviations: keV: kiloelectron volt, O-MAR: orthopedic metal artifact reduction, DL-MAR: deep learning based metal artifact reduction. **Supplemental materials 3B.** *P*-values for pairwise comparisons of Hounsfield units in muscle. *statistically significant after Holm-Bonferroni correction. Abbreviations: keV: kiloelectron volt, O-MAR: orthopedic metal artifact reduction, DL-MAR: deep learning based metal artifact reduction. **Supplemental materials 3C.** *P*-values for pairwise comparisons of Hounsfield units in fat. *statistically significant after Holm-Bonferroni correction. Abbreviations: keV: kiloelectron volt, O-MAR: orthopedic metal artifact reduction, DL-MAR: deep learning based metal artifact reduction. **Supplemental materials 3D.** *P*-values for pairwise comparisons of noise as the standard deviation in Hounsfield units in bladder. *statistically significant after Holm-Bonferroni correction. Abbreviations: keV: kiloelectron volt, O-MAR: orthopedic metal artifact reduction, DL-MAR: deep learning based metal artifact reduction. **Supplemental materials 3E.** *P*-values for pairwise comparisons of noise as the standard deviation in Hounsfield units in muscle. *statistically significant after Holm-Bonferroni correction. Abbreviations: keV: kiloelectron volt, O-MAR: orthopedic metal artifact reduction, DL-MAR: deep learning based metal artifact reduction. **Supplemental materials 3F.** *P*-values for pairwise comparisons of noise as the standard deviation in Hounsfield units in fat. *statistically significant after Holm-Bonferroni correction. Abbreviations: keV: kiloelectron volt, O-MAR: orthopedic metal artifact reduction, DL-MAR: deep learning based metal artifact reduction.

## Data Availability

The datasets and material used during the current study are available from the corresponding author on reasonable request.

## Abbreviations

CNR: Contrast-to-noise ratio
CT: Computed tomography
DL: Deep learning
DL-MAR: DL-based metal artifact reduction
MonoE: Monoenergetic images
O-MAR: Orthopedic metal artifact reduction
ROI: Region of interest
THA: Total hip arthroplasty
