# Supplementary material for: Image quality and metal artifact reduction in total hip arthroplasty CT: deep learning-based algorithm versus virtual monoenergetic imaging and orthopedic metal artifact reduction
Source: Eur Radiol Exp. 2024 Mar 14;8:31. doi: 10.1186/s41747-024-00427-3 (PMC10937891; doi:10.1186/s41747-024-00427-3)
Supplement: Supplementary file 1 — Additional file 1: Supplemental materials 1. Three examples (a-c) of placement of regions of interest (ROI) in the bladder (ROI 1), muscle (ROI 2) and fat (ROI 3). Supplemental materials 2A. P-values for pairwise comparisons of the reconstructed images for image quality. *statistically significant after Holm-Bonferroni correction. Abbreviations: keV: kiloelectron volt, O-MAR: orthopedic metal artifact reduction, DL-MAR: deep learning based metal artifact reduction. Supplemental materials 2B. P-values for pairwise comparisons of the reconstructed images for diagnostic confidence for bone structures.*statistically significant after Holm-Bonferroni correction. Abbreviations: keV: kiloelectron volt, O-MAR: orthopedic metal artifact reduction, DL-MAR: deep learning based metal artifact reduction. Supplemental materials 2C. P-values for pairwise comparisons of the reconstructed images for diagnostic confidence for pelvic organs.*statistically significant after Holm-Bonferroni correction. Abbreviations: keV: kiloelectron volt, O-MAR: orthopedic metal artifact reduction, DL-MAR: deep learning based metal artifact reduction. Supplemental materials 2D. P-values for pairwise comparisons of the reconstructed images for diagnostic confidence for soft tissue adjacent to the prosthesis. *statistically significant after Holm-Bonferroni correction. Abbreviations: keV: kiloelectron volt, O-MAR: orthopedic metal artifact reduction, DL-MAR: deep learning based metal artifact reduction. Supplemental materials 2E. P-values for pairwise comparisons of the reconstructed images for metal artifacts. *statistically significant after Holm-Bonferroni correction. Abbreviations: keV: kiloelectron volt, O-MAR: orthopedic metal artifact reduction, DL-MAR: deep learning based metal artifact reduction. Supplemental materials 3A. P-values for pairwise comparisons of Hounsfield units in bladder. *statistically significant after Holm-Bonferroni correction. Abbreviations: keV: kiloelectron volt, O-MAR: o [file 41747_2024_427_MOESM1_ESM.zip › Supplemental materials 3_ESM.docx]

**Supplemental materials 3**

Supplemental materials 3A: P-values for pairwise comparisons of Hounsfield units in bladder. *statistically significant after Holm-Bonferroni correction. Abbreviations: keV: kiloelectron volt, O-MAR: orthopedic metal artifact reduction, DL-MAR: deep learning based metal artifact reduction.

|  | *Conventional* | *130 keV* | *Conventional + O-MAR* | *130 keV*  *+ O-MAR* | *DL-MAR* |
| --- | --- | --- | --- | --- | --- |
| *Conventional* |  | p = 0.107 | p < 0.001* | p < 0.001* | p < 0.001* |
| *130 keV* |  |  | p = 0.001* | p < 0.001* | p < 0.001* |
| *Conventional + O-MAR* |  |  |  | p = 0.280 | p < 0.001* |
| *130 keV + O-MAR* |  |  |  |  | p < 0.001* |
| *DL-MAR* |  |  |  |  |  |

Supplemental materials 3B: P-values for pairwise comparisons of Hounsfield units in muscle. *statistically significant after Holm-Bonferroni correction. Abbreviations: keV: kiloelectron volt, O-MAR: orthopedic metal artifact reduction, DL-MAR: deep learning based metal artifact reduction.

|  | *Conventional* | *130 keV* | *Conventional + O-MAR* | *130 keV*  *+ O-MAR* | *DL-MAR* |
| --- | --- | --- | --- | --- | --- |
| *Conventional* |  | p = 0.943 | p < 0.001* | p < 0.001* | p < 0.001* |
| *130 keV* |  |  | p = 0.001* | p = 0.001* | p < 0.001* |
| *Conventional + O-MAR* |  |  |  | p = 0.097 | p < 0.001* |
| *130 keV + O-MAR* |  |  |  |  | p < 0.001* |
| *DL-MAR* |  |  |  |  |  |

Supplemental materials 3C: P-values for pairwise comparisons of Hounsfield units in fat. *statistically significant after Holm-Bonferroni correction. Abbreviations: keV: kiloelectron volt, O-MAR: orthopedic metal artifact reduction, DL-MAR: deep learning based metal artifact reduction.

|  | *Conventional* | *130 keV* | *Conventional + O-MAR* | *130 keV*  *+ O-MAR* | *DL-MAR* |
| --- | --- | --- | --- | --- | --- |
| *Conventional* |  | p < 0.001* | p = 0.302 | p = 0.001* | p = 0.220 |
| *130 keV* |  |  | p < 0.001* | p = 0.171 | p = 0.001* |
| *Conventional + O-MAR* |  |  |  | p < 0.001* | p = 0.004* |
| *130 keV + O-MAR* |  |  |  |  | p < 0.001* |
| *DL-MAR* |  |  |  |  |  |

Supplemental materials 3D: P-values for pairwise comparisons of noise as the standard deviation in Hounsfield units in bladder. *statistically significant after Holm-Bonferroni correction. Abbreviations: keV: kiloelectron volt, O-MAR: orthopedic metal artifact reduction, DL-MAR: deep learning based metal artifact reduction.

|  | *Conventional* | *130 keV* | *Conventional + O-MAR* | *130 keV*  *+ O-MAR* | *DL-MAR* |
| --- | --- | --- | --- | --- | --- |
| *Conventional* |  | p = 0.001* | p < 0.001* | p < 0.001* | p < 0.001* |
| *130 keV* |  |  | p = 0.001* | p < 0.001* | p < 0.001* |
| *Conventional + O-MAR* |  |  |  | p < 0.001* | p = 0.118 |
| *130 keV + O-MAR* |  |  |  |  | p = 0.049 |
| *DL-MAR* |  |  |  |  |  |

Supplemental materials 3E: P-values for pairwise comparisons of noise as the standard deviation in Hounsfield units in muscle. *statistically significant after Holm-Bonferroni correction. Abbreviations: keV: kiloelectron volt, O-MAR: orthopedic metal artifact reduction, DL-MAR: deep learning based metal artifact reduction.

|  | *Conventional* | *130 keV* | *Conventional + O-MAR* | *130 keV*  *+ O-MAR* | *DL-MAR* |
| --- | --- | --- | --- | --- | --- |
| *Conventional* |  | p = 0.186 | p < 0.001* | p < 0.001* | p < 0.001* |
| *130 keV* |  |  | p < 0.001* | p < 0.001* | p < 0.001* |
| *Conventional + O-MAR* |  |  |  | p = 0.001* | p < 0.001* |
| *130 keV + O-MAR* |  |  |  |  | p = 0.001* |
| *DL-MAR* |  |  |  |  |  |

Supplemental materials 3F: P-values for pairwise comparisons of noise as the standard deviation in Hounsfield units in fat. *statistically significant after Holm-Bonferroni correction. Abbreviations: keV: kiloelectron volt, O-MAR: orthopedic metal artifact reduction, DL-MAR: deep learning based metal artifact reduction.

|  | *Conventional* | *130 keV* | *Conventional + O-MAR* | *130 keV*  *+ O-MAR* | *DL-MAR* |
| --- | --- | --- | --- | --- | --- |
| *Conventional* |  | p < 0.001* | p < 0.001* | p < 0.001* | p < 0.001* |
| *130 keV* |  |  | p < 0.001* | p < 0.001* | p < 0.001* |
| *Conventional + O-MAR* |  |  |  | p < 0.001* | p < 0.001* |
| *130 keV + O-MAR* |  |  |  |  | p < 0.001* |
| *DL-MAR* |  |  |  |  |  |
